# Development of antibody functionalized magnetic nanoparticles for the immunoassay of carcinoembryonic antigen: a feasibility study for clinical use

**DOI:** 10.1186/s12951-014-0044-6

**Published:** 2014-11-26

**Authors:** Che-Chuan Yang, Shieh-Yueh Yang, Chia-Shin Ho, Jui-Feng Chang, Bing-Hsien Liu, Kai-Wen Huang

**Affiliations:** MagQu Co., Ltd., Sindian Dist, New Taipei City, 231 Taiwan; Department of Surgery & Hepatitis Research Center, National Taiwan University Hospital, Taipei, 100 Taiwan; Graduate Institute of Clinical Medicine, College of Medicine, National Taiwan University, Taipei, 100 Taiwan

**Keywords:** Bio-magnetic nanoparticles, Carcinoembryonic antigen, Assay, Colorectal cancer

## Abstract

**Background:**

Magnetic nanoparticles functionalized antibodies are used for *in*-*vitro* assays on bio-markers. This work demonstrates the synthesis of high-quality magnetic nanoparticles coated with antibodies against carcinoembryonic antigen (CEA). Various characterizations, such as particle size, particle suspension, bio-activity and the stability of bio-magnetic nanoparticles suspended in liquid, are studied. The properties for the assay of CEA molecules in serum are also studied. The assay method used is so-called immunomagnetic reduction.

**Results:**

The results show that the effects of common materials in serum that interfere with detected signals are not significant. The low-detection limit is 0.21 ng/ml, which is well below the clinical threshold of 2.5 ng/ml.

**Conclusions:**

The dynamic range for the assay of CEA molecules in serum is 500 ng/ml. By assaying serum CEA molecules from 24 normal controls and 30 colorectal-cancer patients, the threshold for the serum-CEA concentration to diagnose colorectal cancer is 4.05 ng/ml, which results in a clinical sensitivity of 0.90 and specificity of 0.87.

## Background

Bio-functionalized magnetic particles are used in biomedicines. Different bio-applications require different sizes of magnetic particles. For example, because each particle is strongly magnetized, magnetic particles with micrometer diameters are useful for *in*-*vitro* extraction or purification of bio-molecules such as antibodies, proteins and nucleic acids [1-3]. Magnetic nanoparticles with sub-micrometer diameters are used to sort specific cells *in vitro*, [4-6]. The main reason for the use of sub-micro-particles instead of micro-particles for cell sorting is to suppress the immunological responses from cells that are bound with magnetic particles. Nano-scaled magnetic particles are mostly used for *in*-*vivo* targeting or delivery, e.g. as a contrast medium for magnetic resonance imaging, vectors for drug delivery and for hyperthermia [7-10]. In the late 1990’s, the *in*-*vitro* quantitative detection of bio-molecules using antibody functionalized magnetic nanoparticles was proposed [11-13]. This is referred to as a magnetically labeled immunoassay (MLI).

There are several types of MLI: sandwiched MLI [12,14,15], wash-free MLI [11,13] and single-probe MLI [11,13,16]. Different types of magnetic signals are detected for various types of MLI, including, ac magnetic susceptibility [15], magnetic relaxation [11], magnetic remanence [12], phase lag for ac magnetization [17], nuclear magnetic resonance [18] and magnetic reduction [13] and these are related to the concentrations of bio-molecules that are to be detected. In addition to this academic innovation, the literature shows that MLI is a promising method for *in*-*vitro* diagnosis in clinics. Since the early part of this century, some MLI technology has been commercialized in the US [19], France [20], Germany, Sweden [21], Japan, China [22] and Taiwan [23]. There has been continuing investment in the development, the commercialization and the marketing of MLI, worldwide.

In a MLI, bio-functionalized magnetic nanoparticles are used as labeling markers to target molecules. If a test sample has more target molecules, more magnetic nanoparticles associate with target molecules. Ideally, each magnetic nanoparticle is identical. Every nanoparticle has the same size and magnetization. Each associated magnetic nanoparticle contributes equally to the magnetic signals. If more magnetic nanoparticles associate with target molecules, the magnetic signal is greater. The magnetic signals are exactly correlated to the number of target molecules. The precession of assay target molecules is high. However, if the magnetic nanoparticles obviously differ from each other and there is a broad variation in particle size, magnetic nanoparticles of different sizes contribute differently to the magnetic signals. This results in a significant variation in the magnetic signals for a fixed number of associated magnetic nanoparticles, so the precision the assay of target molecules is poor. For a MLI, it is important that the bio-functionalized magnetic nanoparticles are uniform. For a MLI, magnetic nanoparticles are suspended in solution as a reagent. When these nanoparticles agglomerate, the binding area between the nanoparticles and the target molecules is significantly reduced, which results in a reduced sensitivity and stability for detection, so the agglomeration of nanoparticles in a reagent must be inhibited.

Other required properties for the use of suspended bio-functionalized magnetic nanoparticles as a reagent for *in*-*vitro* diagnosis in clinics are the life time, the interference, the low-detection limit, the sensitivity and the specificity. Most previous studies have focused on the development of either magnetic nanoparticles or detection methodologies, so there has been no complete study of the feasibility of the clinical use of bio-functionalized magnetic nanoparticles for *in*-*vitro* diagnosis. This study characterizes both the particle properties and the assay features of antibody functionalized magnetic nanoparticles. The target molecule is the carcinoembryonic antigen (CEA), which is the clinical bio-marker for the *in*-*vitro* diagnosis of colorectal cancer. The antibodies against CEA (anti-CEA) are immobilized on magnetic nanoparticles. Various characteristics, such as particle size, particle suspension, bio-activity and the stability of the anti-CEA functionalized magnetic nanoparticles suspended in liquid are studied. The assay method used is the so-called immunomagnetic reduction. Assaying CEA in serum allows features such as the interference, the low-detection limit, the dynamic range, the clinic sensitivity and the specificity to be determined.

## Results and discussion

### Stability of magnetic nanoparticle suspension

**Figure 1:**
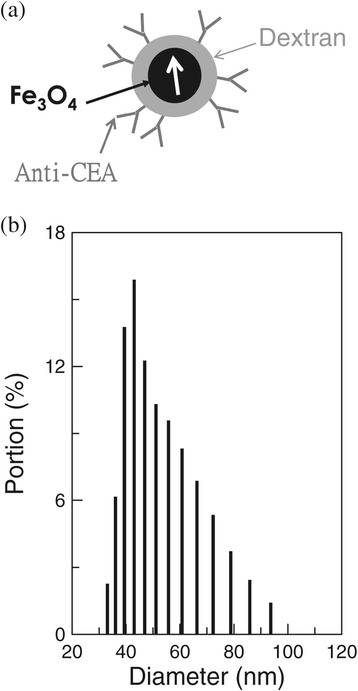
**Anti-CEA functionalized magnetic nanoparticle.** The schematic composition and the distribution of the diameter of anti-CEA functionalized magnetic nanoparticles are shown in **(a)** and **(b)**, respectively.

**Figure 2:**
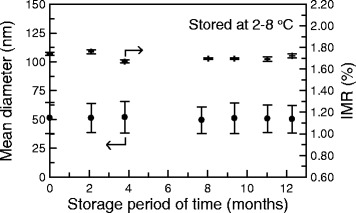
**Stability tests, in terms of the mean diameter (•) and the bio-activity (✝) of anti-CEA functionalized magnetic nanoparticles dispersed in PBS solution, stored at 2–8°C.**

The schematic composition of anti-CEA functionalized magnetic nanoparticles is shown in Figure 1(a). The distribution of anti-CEA functionalized magnetic nanoparticles suspended in PBS solution in hydrodynamic diameter is shown in Figure 1(b). The mean value and the standard deviation of the hydrodynamic diameter are found to be 51.3 nm and 13.51 nm, respectively, as measured using dynamic laser scattering. Hereafter, the anti-CEA functionalized magnetic nanoparticles suspended in PBS solution are referred to as CEA reagent. The CEA reagent was stored at 2–8°C. During the storage, the mean value and standard deviation for the hydrodynamic diameter of anti-CEA functionalized magnetic nanoparticles were monitored. The results are shown in Figure 2, as dots with error bars. The error bars correspond to the standard deviation of the hydrodynamic diameter of anti-CEA functionalized magnetic nanoparticles. It is obvious that the particle diameter remains almost unchanged, when it is stored at 2–8°C for 12 months. This shows that there is no significant agglomeration of the anti-CEA functionalized magnetic nanoparticles in CEA reagent that is stored at 2–8°C for 12 months. Namely, the suspension of anti-CEA functionalized magnetic nanoparticles remains stable for 12 months. The stability of the suspension of the nanoparticles in the reagent is important for clinic use. When nanoparticles agglomerate, the association area between antibodies and target molecules varies. The output signal decays as the association area is reduced. The assay result is unreliable, if there is any agglomeration of nanoparticles. Fortunately, the results shown as dots in Figure 2 demonstrate the high stability of the suspension of anti-CEA functionalized magnetic nanoparticles in PBS solution, stored at 2–8°C.

The factors that mainly affect the stability of a nanoparticle suspension in solution are the nano-size and the uniformity of the nanoparticles. If the nano-size is sufficient, the buoyancy of the nanoparticle is strong enough to cancel the gravitational force, so the nanoparticle is suspended in solution. However, for a certain magnetic nanoparticle in the reagent, there can be attractive or repulsive magnetic interactions between the magnetic nanoparticle and neighboring magnetic nanoparticles. If the magnetic nanoparticles are highly uniform, these magnetic interactions are isotropic. The resultant magnetic force that acts on this magnetic nanoparticle is zero and the magnetic interactions with these magnetic nanoparticles can be ignored. This nanoparticle is not attracted by other particles because there are no anisotropic magnetic forces. Magnetic nanoparticles do not agglomerate because of the isotropic magnetic interaction between particles, so highly uniform magnetic nanoparticles exhibit high stability in suspension in solution. The results shown in Figure 1(b) show the nano-size and the high degree of uniformity of the anti-CEA functionalized magnetic nanoparticles synthesized in this work. As a result, the CEA reagent is highly stable in a suspension in PBS solution.

### Stability of magnetic nanoparticle bio-activity

In addition to the suspension stability, the other important measurement for the CEA reagent is the bio-activity during storage at 2–8°C. To measure this, the IMR signals for 5-ng/ml CEA solution were detected using the CEA reagent, during the storage period. The measurements for the storage period for the IMR signals for the 5-ng/ml CEA solution are plotted in Figure 2, using crosses. The IMR signals range from 1.67% to 1.76%, during storage at 2–8°C for nine months. There is no significant change in the IMR signal for the 5-ng/ml CEA solution during nine months of storage at 2–8°C. Therefore, the bio-activity of the CEA reagent is stable for at least nine months, if the CEA reagent is stored at 2–8°C.

### Room-temperature stability of reagent

**Figure 3:**
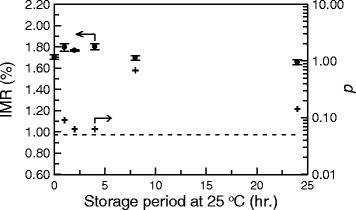
**Stability tests for the bio-activity of anti-CEA functionalized magnetic nanoparticles dispersed in PBS solution, stored at 25°C using immunomagnetic reduction.** The *p* values for the IMR signals with respect to the initial IMR signals are plotted.

For the experimental results shown in Figure 2, the CEA reagent was originally at 2–8°C and was then stored at a room temperature of 25°C. The temperature of the CEA reagent was gradually increased from 2–8°C to 25°C over 5 minutes. 40-μl CEA reagent was used for each IMR measurement. The remainder of the CEA reagent was replace in storage at 2–8°C. When all of the samples were ready, the stored CEA reagent was warmed to 25°C, from 2–8°C, for IMR measurements. This thermal circle could easily damage the bio-activity of CEA reagent, so the CEA reagent was maintained at 25°C, to allow continuous IMR measurements for several samples. In order to determine the bio-activity of CEA reagent stored at 25°C, the CEA reagent was moved from a storage temperature of 2–8°C to 25°C. After 5 minutes, the temperature of the CEA reagent reached 25°C and the CEA reagent was maintained at 25°C for 24 hours. The time period in the x axis in Figure 3 begins at the 5^th^ minute after the reagent was moved from a storage temperature of 2–8°C to 25°C. The signals for 5-ng/ml CEA solutions for the 25-°C CEA reagent were detected for 24 hours. The results are plotted as dots in Figure 3. The IMR signals at the beginning, i.e. time period =0, are used as a reference. The *p* values of the IMR signals at other time points with respect to the referenced IMR signal are plotted as crosses in Figure 3. All of the *p* values are greater than 0.05, which means that there is no significant difference in the IMR signals shown in Figure 3. The results in Figure 3 show that the bio-activity is stable for 24 hours, even if the CEA reagent is stored at 25°C.

### Interference tests

In clinics, serum is the sample that is used to assay CEA. There are many other materials in serum, besides CEA. These materials are referred as to interfering materials. Interfering materials can cause false IMR signals, because of the colors of bio-molecules or the non-specific associations between the interfering materials and antibodies on the magnetic nanoparticles. The accuracy of an assay is adversely affected if false IMR signals occur frequently, so the contributions of the interfering materials to IMR signals for the assay of CEA in serum must be determined.

Serum can contain interfering materials such as hemoglobin, bilirubin, or triglyceride because of common diseases, such as hemolysis, jaundice or hypertriglyceridemia. Other bio-materials that exist naturally in serum, such as uric acid, rheumatoid factor, intra lipid or albumin, are also interfering materials. Other interfering materials include drugs or chemicals in medicine that is used to treat inflammatory diseases, viral and bacteria infections, cancers and cardiovascular disease. All of the natural bio-materials and drugs or chemicals tabulated in Table 1 were spiked into serum that had 5 ng/ml CEA. A 5-ng/ml CEA solution was used because this CEA concentration is approximately the clinical threshold of CEA concentration for the diagnosis of colorectal cancer (~2.5 to 5 ng/ml). The concentrations of these interfering materials are also listed in Table 1. It is worthy of note that the concentrations of the interfering materials are much greater than ordinary levels. For example, the level of hemoglobin in the blood of a patient with hemolysis is around 500 μg/ml. The concentration of hemoglobin used in Sample No. 2 is 1000 μg/ml. The IMR signals for these 5-ng/ml CEA serum solutions are listed in Table 1. The IMR signal for the serum (Sample No. 1) with only 5-ng/ml CEA is used as a reference. All of the other IMR signals for the serum samples (Sample Nos. 2–32) with both 5-ng/ml CEA and the interfering materials are compared with the reference IMR signal. The corresponding *p* values calculated T-Test and are shown in Table 1. The *p* values IMR signals for the serum samples with interfering materials are greater than 0.05, as shown in Table 1. This demonstrates that, with the exception of acetyl cysteine and furosemide, the bio-molecules, drugs and chemicals listed in Table 1 do not interfere with the assay for CEA in serum.

**Table 1:** **Materials and concentrations used for interference tests for a CEA assay**, **using the IMR method**

**Sample no.**	**Interfering material**	**Concentration**	**Mean IMR value** **(%)**	**Standard deviation of IMR** **(%)**	***p*** **value**
1	None	-	1.70	0.021	-
2	Hemoglobin	10000 μg/ml	1.71	0.014	0.246
3	Bilirubin	600 μg/ml	1.66	0.021	0.100
4	Triglyceride	30000 μg/ml	1.72	0.007	0.167
5	Uric acid	200 μg/ml	1.69	0.021	0.150
6	Rheumatoid factor	500 IU/ml	1.68	0.014	0.246
7	Intra lipid	30000 μg/ml	1.67	0.007	0.099
8	Albumin	60000 μg/ml	1.67	0.014	0.150
9	Acetaminophen	300 μg/ml	1.72	0.007	0.167
10	Acetyl cysteine	150 μg/ml	1.68	0.021	0.223
11	Acetylsalicylic acid	500 μg/ml	1.74	0.021	0.100
12	Ascorbic acid	300 μg/ml	1.71	0.014	0.246
13	Atrovastatin	3 μg/ml	1.71	0.014	0.246
14	Furosemide	4000 μg/ml	1.70	0.014	0.404
15	Ibuprofen	1000 μg/ml	1.72	0.049	0.326
16	Levodopa	20 μg/ml	1.71	0.014	0.246
17	Methyldopa	200 μg/ml	1.72	0.014	0.150
18	Naprosyn sodium	500 μg/ml	1.66	0.021	0.100
19	Phenylbutazone	400 μg/ml	1.65	0.007	0.052
20	Prednisone	5 μg/ml	1.69	0.014	0.404
21	Tegafur with uracil	50 μg/ml	1.70	0.021	0.500
22	Theophylline	50 μg/ml	1.69	0.014	0.404
23	Warfarin	50 μg/ml	1.66	0.014	0.096
24	Ampicillin sodium	1000 μg/ml	1.67	0.021	0.146
25	Cefoxitin	2500 μg/ml	1.73	0.014	0.096
26	Cyclosporeine A	10 μg/ml	1.71	0.007	0.296
27	Doxycycline hyclate	50 μg/ml	1.68	0.021	0.223
28	Irinotecan	100 μg/ml	1.69	0.014	0.404
29	Lovastatin	2.5 μg/ml	1.72	0.014	0.150
30	Metronidazole	200 μg/ml	1.70	0.021	0.500
31	Oxaliplatin	100 μg/ml	1.72	0.014	0.150
32	Rifampicin	60 μg/ml	1.74	0.007	0.051

The concentration of the CEA in each sample is 5 ng/ml. The matrix is serum. The detected mean value and the standard deviation of each sample are listed. The *p* values of IMR signals for other samples are calculated using the IMR signals for the pure CEA-serum sample as a reference and the results are listed in the right-most column.

The false IMR signal that is caused by interfering materials is mainly attributable to two factors: the color of the interfering materials and the non-specific associations between the interfering materials and antibodies on the magnetic nanoparticles. The detection signal used for IMR measurement is magnetic ac susceptibility, which is not affected by the colors of the samples, the reagents, or the interfering materials, so the color of the interfering materials does not cause false IMR signals to be generated.

The non-specific associations between the interfering materials and antibodies on the magnetic nanoparticles are inhibited if highly specific antibodies are used. An additional suppression mechanism is activated during IMR measurement. During the IMR measurement, the magnetic nanoparticles oscillate with the external ac magnetic fields. Both the target and other bio-molecules that are bound with antibodies on the oscillating magnetic nanoparticles experience centrifugal forces. At high oscillation frequencies, the centrifugal force is increased. If the centrifugal force is stronger than the binding force between the antibodies and non-target bio-molecules, the non-specific binding is broken, so the centrifugal force must be weaker than the specific binding force between the antibodies and the bio-molecules that are to be detected. As a result, the cross reactions are inhibited during IMR measurement. In principle, this suppression mechanism for non-specific binding between antibodies and non-target bio-molecules is independent of the concentration of the target molecules, such as CEA. A detailed discussion of this suppression mechanism is given in Ref. [24].

### CEA-concentration dependent IMR signals

**Figure 4:**
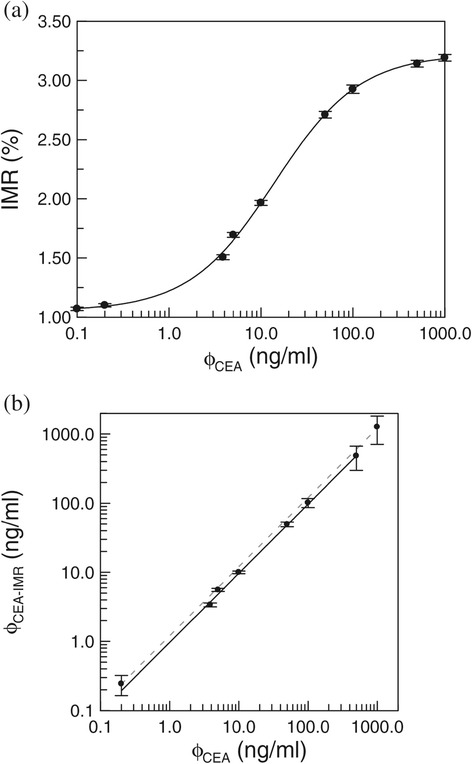
**Spiked-CEA-concentration-in-serum, ϕ**_**CEA**_ **, and (a) IMR signal and (b) the CEA concentration, ϕ**_**CEA-IMR**_ **, derived using the immunomagnetic reduction method.**

In addition to the detection of IMR signal for the 5-ng/ml CEA serum sample, the IMR signals for serum samples with CEA at various concentrations, from 0.1 ng/ml to 1000 ng/ml, were measured. The experimental results for the relationship between the CEA-concentration and the IMR signal (IMR(%)-ϕ_CEA_) are plotted as dots in Figure 4(a). The detailed results are tabulated in Table 2. The error bar for each data point in Figure 4(a) corresponds to the standard deviation for multiple detections of IMR signals for a given test sample. The IMR signal gradually increases, as the CEA concentration increases from 0.1 ng/ml, and then almost becomes saturated at a CEA concentration of 500 ng/ml. The IMR(%)-ϕ_CEA_ relationship is described by the logistic function:

**Table 2:** **CEA concentrations**, ϕ_**CEA**_, **spiked in serum**, **for the detections of IMR signals**

ϕ_**CEA**_ **(** **ng** **/** **ml** **)**	**Mean IMR value** **(%)**	**Standard deviation of IMR** **(%)**	**Converted CEA concentration** ϕ_**CEA**-**IMR**_ **(** **ng** **/** **ml** **)**
0.10	1.07	0.014	0.08 ± 0.07
0.20	1.10	0.014	0.24 ± 0.08
3.85	1.51	0.021	3.38 ± 0.21
5.00	1.70	0.021	5.58 ± 0.28
10.0	1.97	0.021	9.99 ± 0.43
50.0	2.71	0.028	49.49 ± 3.83
100	2.93	0.035	101.7 ± 15.0
500	3.14	0.028	482.6 ± 184.7
1000	3.19	0.028	1269.6 ± 560

The mean values and the standard deviations of the detected IMR signals are shown. Using the detected IMR signals, the CEA concentrations, ϕ_CEA-IMR_, derived using Eq. (1), are listed in the right-most column.

1 $$ IMR\left(\%\right)=\frac{A-B}{1+{\left(\frac{\varphi_{CEA}}{\varphi_o}\right)}^{\gamma }}+B $$

where A, B, ϕ_o_ and γ are fitting parameters. Fitting the data in Figure 4(a) to Eq. (1) gives values for these parameters of as A =1.05, B =3.22, ϕ_o_ =14.05, and γ =0.94. The fitting curve is plotted as a solid line in Figure 4(a). The coefficient of determination, R^2^, is 0.999.

The stability of the resulting logistic function Eq. (1) depends on the stability of the reagent. Several factors, such as the biodegradability of the antibodies on the magnetic nanoparticles, the agglomeration of the magnetic nanoparticles and the de-magnetization of the magnetic nanoparticles significantly affect the lifetime of the reagent. If these factors do not change, the characteristics of the logistic function Eq. (1) are retained.

In Figure 2, the high stability of the particles’ suspension and isolation in the reagent is demonstrated. The particle size is plotted as a function of the storage time at 2–8°C. The IMR signal for 5-ng/ml CEA solution is dependent on storage time, which demonstrates the bio-active stability of the antibodies on the magnetic nanoparticles. The magnetization of the magnetic nanoparticles does not change if the storage temperature remains lower than the Curie temperature of Fe_3_O_4_ (~585°C). The results in Figure 2 show that the factors that most greatly affect the lifetime of the reagent are unchanged, if the reagent is stored at 2–8°C for 12 months, so it is expected that the resultant logistic function Eq. (2) is stable over 12 months.

### Low-detection limit of assaying serum CEA

In Eq. (1), A denotes the IMR signal as the CEA concentration approaches zero, so parameter A in Eq. (1) represents the background level for the detection of IMR signals. This non-zero background level is mainly due to the electronic noise of the IMR analyzer and the dynamic equilibrium for the association between the CEA molecules and anti-CEA on the magnetic nanoparticles. Using the 3-σ criterion, the low-detection limit for the assay of CEA in terms of the IMR signal is (1.05 + 3 × 0.014)% =1.09%, where 0.014% is the standard deviation of the IMR signals at low CEA concentrations, such as 0.1 ng/ml. Using Eq. (1), the CEA concentration that corresponds to the IMR signal of 1.09% is 0.21 ng/ml. Therefore, the low-detection limit for the assay of CEA in serum using IMR is 0.21 ng/ml. It is significant that the threshold of CEA concentration in serum for the diagnosis of colorectal cancer is 2.5 ng/ml. The IMR method allows sufficiently sensitive blood tests for the *in*-*vitro* diagnosis of colorectal cancer.

### Linearity of assaying serum CEA

A careful inspection for Figure 4(a) shows that not every data point lies on the solid line. If the experimental IMR signals are used to derive the CEA concentration, using Eq. (1), the derived CEA concentrations, ϕ_CEA-IMR_, are not exactly the same as those ϕ_CEA_ values that are shown in Table 2. It is noted that ϕ_CEA_ denotes the spiked CEA concentration in serum, but ϕ_CEA-IMR_ denotes the CEA concentration in serum, detected using the IMR method. The correlation between ϕ_CEA-IMR_ and ϕ_CEA_ is shown in Figure 4(b). Since the low-detection limit for the assay of CEA using IMR method is 0.21 ng/ml, a ϕ_CEA-IMR_ of 0.1-ng/ml CEA solution is not used in Figure 4(b). Figure 4(b) shows the linear relationship between ϕ_CEA-IMR_ and ϕ_CEA_. If the ϕ_CEA-IMR_ values for CEA concentration ϕ_CEA_ values from 0.2 ng/ml to 1000 ng/ml are used in Figure 4(b), the slope of the ϕ_CEA-IMR_-ϕ_CEA_ curve is 1.21 and the coefficient of determination, R^2^, is 0.989, as plotted with the dashed line in Figure 4(b). The US Food and Drug Administration (FDA) regulations state that the slope of the line in Figure 4(b) must be between 0.90 and 1.10. The slope of the dashed line in Figure 4(b) does not meet the requirement of the US FDA. However, if the ϕ_CEA-IMR_ for a CEA concentration ϕ_CEA_ of 1000 ng/ml is ignored, the curve for ϕ_CEA-IMR_ against ϕ_CEA_ is linear and is plotted with a solid line in Figure 4(b). The slope of this solid line is 0.97 and the coefficient of determination, R^2^, is 0.999. It is worthy of note that the slope of the solid line meets the requirement of the US FDA. The range of CEA concentrations used for the solid line in Figure 4(b) is from 0.2 ng/ml to 500 ng/ml, so the dynamic range of the CEA concentration for IMR assay is 500 ng/ml.

A comparison of the low-detection limit and the dynamic range of a CEA assay for commercially available kits (e.g. Siemens, Abbott, and Roche) used in the work is listed in Table 3. It is clear that the IMR assay for CEA is highly sensitive and has a broad dynamic range, which renders it suitable for clinical use.

**Table 3:** **Comparison of the low**-**detection limit and the dynamic range of a CEA assay using commercially available kits** (**e.g. Siemens**, **Abbott**, **and Roche**) **and those for this work** (**denoted as this work**)

**Company** **/** **Model**	**Low** **-** **detection limit**	**Dynamic range**	**Approval**
Siemens/ADVIA Centuar CEA assay	0.5 ng/ml	0.5 - 100 ng/ml	USFDA
Abbott/AXSYM system CEA assay	0.5 ng/ml	0.5 - 500 ng/ml	USFDA
Roche/Elecsys CEA assay	0.2 ng/ml	0.2 - 1,000 ng/ml	USFDA, CE
This work	0.21 ng/ml	0.21 - 500 ng/ml	None

### Clinical tests for assaying serum CEA

**Figure 5:**
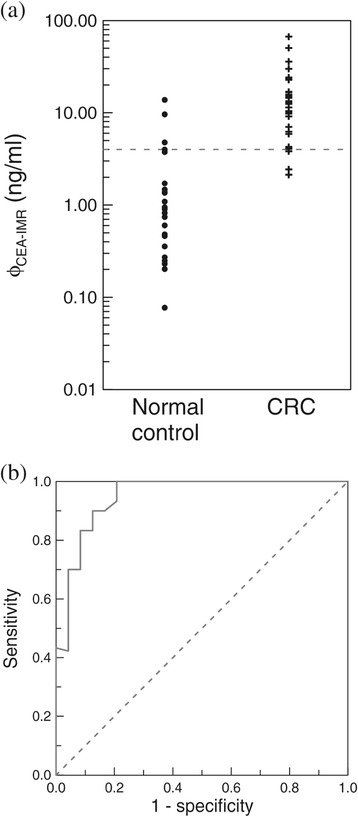
**Comparison of serum CEA concentration for normal controls and CRC. (a)** CEA concentration, ϕ_CEA-IMR_, for normal subjects (normal control) and patients with colorectal cancer (CRC), detected using the immunomagnetic reduction method, and **(b)** the ROC curve for the analysis of the clinical sensitivity and the specificity for the diagnosis of colorectal cancer, using the detected CEA concentration ϕ_CEA-IMR_ in serum.

Using the relationship shown in Figure 4(a), the CEA concentrations in human serum samples can be determined using the IMR signals. 24 serum samples from healthy subjects (Normal control) and 30 serum samples from patients with colorectal cancer (CRC) were used for the CEA assay, using the IMR method. CRC patients were identified using either pathological evidence or an immunoassay. The detected CEA concentrations ϕ_CEA-IMR_ of these serum samples are plotted in Figure 5(a). The ϕ_CEA-IMR_ values for the normal control group are distributed over a relatively lower range than those for CRC patients. Most of ϕ_CEA-IMR_ values for the normal control group are within the range, 0.6 ng/ml to 1.5 ng/ml, but the ϕ_CEA-IMR_ values for CRC patients range from 6.0 ng/ml to 20 ng/ml. An analysis of the receiver operating characteristic (ROC) curve shown in Figure 5(b) shows that the threshold for the diagnosis of CRC by an assay of CEA in serum, using the IMR method, is 4.05 ng/ml, which results in the clinic sensitivity of 0.90 and a specificity of 0.87.

## Conclusions

Antibodies against carcinoembryonic antigen (CEA) are conjugated onto magnetic nanoparticles, to synthesize a magnetic reagent for the assay of CEA in serum. The reagent gives a highly stable particle suspension in pH-7.4 phosphate buffered saline (PBS) solution and CEA molecules with a highly stable bio-activity, when the reagent is stored at 2–8°C. The immunomagnetic reduction method gives a low-detection threshold for the assay of CEA is 0.21 ng/ml and the dynamic range is 500 ng/ml. There is no significant interference with the assay of CEA in serum by bio-molecules that commonly occur in serum, or by frequently used drugs or chemicals. The results for CEA concentration in 54 serum samples show that the clinical sensitivity and the specificity for the diagnosis of colorectal cancer are 0.90 and 0.87, respectively, with a threshold of 4.05 ng/ml.

## Methods

### Synthesis of bio-magnetic nanoparticles

The protocol for the synthesis of magnetic Fe_3_O_4_ nanoparticles is proposed by MagQu Co., Ltd [25]. A ferrite solution containing ferrous sulphate hepta-hydrate (FeSO_4_⋅7H_2_O) and ferric chloride hexa-hydrate (FeCl_3_⋅6H_2_O) in a stoichiometric ratio of 1:2 was mixed with an equal volume of aqueous dextran, which acts as a surfactant for Fe_3_O_4_ particles dispersed in water. The mixture was heated to 70–90°C and titrated with a strong base solution, to form black Fe_3_O_4_ particles. Aggregates and excess unbound dextran were removed by centrifugation and gel filtration chromatography, to produce a highly concentrated homogeneous magnetic fluid. The reagent (MF-DEX-0060, MagQu) with the desired magnetic concentration was obtained by diluting the highly concentrated magnetic fluid with pH-7.4 phosphate buffered saline (PBS) solution. To ensure that the antibodies against carcinoembryonic antigen (CEA) for colorectal cancer, i.e. anti-CEA (AT-CEA, MagQu), bound to the dextran on the outmost shell of magnetic nanoparticles, NaIO_4_ solution was added into the magnetic solution to oxidize the dextran, which then creates aldehyde groups (−CHO). The dextran then reacts with anti-CEA via the linking of –CH = N-, so anti-CEA is bound covalently to dextran, as schematically shown in Figure 1(a). Unbound anti-CEA was separated from the solution by magnetic separation.

### Characterizations of bio-magnetic nanoparticles

The distribution of the size of Fe_3_O_4_ magnetic nanoparticles bio-functionalized with anti-CEA was determined by dynamic laser scattering (Nanotrac 150, Microtrac). To determine the immuno reactivity of anti-CEA functionalized Fe_3_O_4_ magnetic nanoparticles, so-called immunomagnetic reduction was used [13,26,27]. In immunomagnetic reduction, antibody functionalized magnetic nanoparticles were mixed with a solution of target bio-molecules. Before the nanoparticles and CEA molecules associated, the ac magnetic susceptibility χ_ac,o_ of the mixture was detected, using an ac magnetosusceptometer (XacPro-E, MagQu). Nanoparticles associate with target bio-molecules, via the antibodies on the magnetic nanoparticles. This association results in a reduction in the ac magnetic susceptibility of the mixture. The ac susceptibility of the mixture after association is denoted by χ_ac,ϕ_. The reduction in ac susceptibility is referred to as the IMR signal and is expressed as:

2 $$ \mathrm{I}\mathrm{M}\mathrm{R}\left(\%\right) = \left[\left({\chi}_{\mathrm{ac},\mathrm{o}}-{\chi}_{\mathrm{ac},\varphi}\right)/{\chi}_{\mathrm{ac},\mathrm{o}}\right]\times 100\% $$

For each IMR signal measurement, 40-μl reagent and 60-μl CEA solution or sample were used.

According to Ref. [28], the total number of antibody functionalized magnetic nanoparticles in 1-ml, 8-mg-Fe/ml magnetic bio-reagent is roughly 10^13^ particles. There are averagely 6 antibodies immobilized on one magnetic nanoparticle. For IMR measurement, the used volume of reagent is 40 μl. Hence, 2.4 × 10^12^ anti-CEA molecules are used for each IMR test.

### Preparation of standard CEA solutions

Standard CEA (Human CD66e) protein was obtained from AdD Serotec (Cat. No. PHP282). Various concentrations of CEA protein were prepared by serially diluting standard CEA protein with PBS buffer or normal free serum and the resulting solution was stored at −80°C. The concentration of CEA protein was then quantified using a spectrophotometer (NanoDrop2000; Thermo Scientific.).

### Assessment of human serum CEA

24 serum samples from healthy subjects (Normal control) and 30 serum samples from patients with colorectal cancer (CRC) were used for the CEA assay. The CEA concentrations in human serum samples will be determined using the IMR signals. CRC patients were identified using either pathological evidence or an immunoassay. All of the enrolled patients provided informed consent before undergoing the procedure and this study was approved by National Taiwan University Hospital Research Ethics Committee (No.201105996RC).
